# A Clinical Risk Assessment of a 3D-Printed Patient-Specific Scaffold by Failure Modes and Effects Analysis

**DOI:** 10.3390/ma15155442

**Published:** 2022-08-08

**Authors:** Ping Qi Lim, Sue Huey Lim, Maria Sherilyn, Tulio Fernandez-Medina, Sašo Ivanovski, Sepanta Hosseinpour

**Affiliations:** 1 School of Dentistry, The University of Queensland, Brisbane 4006, Australia; 2 College of Medicine and Dentistry, James Cook University, Cairns Campus, Cairns 4870, Australia

**Keywords:** bone substitutes, scaffold, alveolar bone grafting, guided tissue regeneration, additive manufacturing, risk analysis, FMEA

## Abstract

This study aims to carry out a risk assessment to identify and rectify potential clinical risks of a 3D-printed patient-specific scaffold for large-volume alveolar bone regeneration. A survey was used to assess clinicians’ perceptions regarding the use of scaffolds in the treatment of alveolar defects and conduct a clinical risk assessment of the developed scaffold using the Failure Modes and Effects Analysis (FMEA) framework. The response rate was 69.4% with a total of 41 responses received. Two particular failure modes were identified as a high priority through the clinical risk assessment conducted. The highest mean Risk Priority Number was obtained by “failure of healing due to patient risk factors” (45.7 ± 27.7), followed by “insufficient soft tissue area” (37.8 ± 24.1). Despite the rapid developments, finding a scaffold that is both biodegradable and tailored to the patient’s specific defect in cases of large-volume bone regeneration is still challenging for clinicians. Our results indicate a positive perception of clinicians towards this novel scaffold. The FMEA clinical risk assessment has revealed two failure modes that should be prioritized for risk mitigation (safe clinical translation). These findings are important for the safe transition to in-human trials and subsequent clinical use.

## 1. Introduction

Large bony defects may develop in the alveolar bone of the maxilla and mandible as a consequence of tooth loss, trauma, infection, or tumour resection [1]. In addition, extensive tooth loss caused by dental caries and/or periodontitis are globally prevalent conditions which lead to large alveolar defects [2,3,4]. Loss of tooth-supporting bone predisposes patients to poor dietary intake, lowered productivity, and tremendous financial burden that ultimately impairs quality of life [5,6,7,8]. The high prevalence and impactful nature of this disease at both individual and societal levels means that effective management of bony defects is essential.

In instances where the amount of bone loss exceeds the natural regenerative capacity of bone, treatment of bony defects seeks to reconstruct or reproduce the lost tissues to restore their form and function. However, current regenerative technologies—for example, block bone grafts, Guided Bone Regeneration (GBR) barrier membrane, or 3D-printed titanium scaffolds—still have numerous drawbacks [9]. These issues include donor-site morbidity, high variability in outcome, and limitation of use to only small confined defects [9]. Therefore, regeneration is still unpredictable for large bony and no-wall defects.

To fulfil this unmet treatment need, researchers at the Centre for Orofacial Regeneration, Reconstruction, and Rehabilitation (COR3) (School of Dentistry, The University of Queensland) developed a biodegradable 3D-printed scaffold. This scaffold is a three-dimensional, additively manufactured, highly porous structure made from a medical grade biodegradable polymer (polycaprolactone (PCL)) [10]. Unlike conventional GBR and block bone substitutes, the COR3 scaffold is patient-specific and designed from a computed tomography (CT) scan of the patient’s bone defect [10]. This optimizes adaptation of the scaffold to the defect, hence addressing the issues of handling and fixation. A major advantage over currently available products, including 3D-printed titanium scaffold technology Yxoss (Yxoss CBR^®^, Geistlich Pharma AG, Wolhusen, Switzerland), is that the COR3 scaffold is made of a resorbable polymer that does not require a second surgery for removal and has been safely used in several regulated devices [11]. The porosity of the COR3 scaffold is also designed to allow optimal vascularization for bone regeneration. Its dimensional stability provides space maintenance that prevents collapse of overlying tissue into the defect, which is usually an issue in regenerating large non-confined bony defects. In addition, the scaffold helps to exclude ingrowth of undesirable tissues and allows bone cells to populate the defect instead. 

The preclinical in vitro and animal studies have shown promising results in terms of the efficacy of this product in regenerating dimensionally stable bone extra-skeletally (vertical bone augmentation) [10]. Clinical trials are the next step to ensure consistency between animal models and humans, since there may be interspecies differences in terms of anatomy, healing processes, and host responses. However, it is first necessary to overcome certain ‘regulatory hurdles’ before proceeding to the human clinical trial phase. 

These regulatory requirements are a necessary check-and-balance imposed by regulatory agencies, such as US’s Food and Drug Administration (FDA).,The European Union Medical Device Regulation (EU MDR 2017/745), or Australia’s Therapeutic Goods Administration (TGA) (Table 1), which oversee the safety and quality of therapeutic goods to protect the wellbeing of consumers. Medical devices are classified according to the level of harm they may pose to users or patients. Therefore, one of the first steps, when engaging with regulatory authorities is to find the right classification for a given medical device. This has a direct bearing on the level of regulatory scrutiny which can be expected to ensure all is being done to ensure the safety of the device. For a therapeutic good to be lawfully supplied in Australia, the TGA must first approve its inclusion in the Australian Register of Therapeutic Goods (ARTG). The TGA regulates medical devices in accordance with:

- The Therapeutic Goods Act (1989)
- The Therapeutic Goods (Medical Devices) Regulations 2002
- The Therapeutic Goods Regulations 1990

As part of the conformity assessment process, the manufacturer is required to provide evidence of compliance with the TGA’s Essential Principles, one of which is demonstrating that safety standards in design and construction have been followed to minimize risks.

Reasonably, a systematic method of identifying, evaluating, and controlling potential risks is necessary to minimize the severity and likelihood of harm to users of the product. The COR3 scaffold would be classified by the TGA as a ‘Class III medical device’, defined by Section 41BD of the Therapeutic Goods Act 1989 as any appliance that is used by human beings for the “diagnosis, prevention, monitoring, prediction, prognosis, treatment or alleviation of disease” [12]. Reasonably, a systematic method of identifying, evaluating, and controlling potential risks is necessary to minimize the severity and likelihood of harm to users of the product. The COR3 scaffold would be classified by the EU MDR 2017/745 and TGA as a ‘Class III medical device’, defined by Section 41BD of the Therapeutic Goods Act 1989 as any appliance that is used by human beings for the “diagnosis, prevention, monitoring, prediction, prognosis, treatment or alleviation of disease” [12].

ISO 14971:2019 Application of Risk Management to Medical Devices and its companion document, ISO/TR 24971:2020 Medical devices–Guidance on the application of ISO 14,971 is key for the development, implementation, and maintenance of a risk management system for medical devices [13].

The risk management process consists of six steps, including risk analysis, risk evaluation, risk control, evaluation of overall residual risk, risk management review, and production and post-production activities as described in ISO 14971:2019 Risk analysis is the initial stage and involves the identification of hazards and estimation of risk [13]. A Failure Modes and Effects Analysis (FMEA) is a very useful approach for undertaking a safety analysis for regulatory purposes. IEC 60812: 2018 describes a procedure for planning, performing, documenting, and maintaining an FMEA [14]. It is a bottom-up approach wherein all the ways in which the product could possibly fail are identified, then the causes and effects of each mode of failure are determined [15]. FMEA provides a systematic approach to prioritizing risks in complicated environments such as the biomedical industry, highlighting unacceptable critical failure modes that need to be addressed prior to product use [16]. The C-arm X-ray machine and intrathecal drug therapy are examples of the many biomaterials, medical devices, and therapies that are widely regarded as safe by virtue of gaining an appreciation of device failure modes and associated effects through FMEA [17,18].

Therefore, the aims of this study were to: (i) assess clinicians’ perceptions regarding the current use of scaffolds in the treatment of large-volume alveolar bony defects and (ii) conduct a survey-based clinical risk assessment of the COR3 scaffold using the FMEA framework.

## 2. Materials and Methods

### 2.1. Survey Design

An online survey was developed using Google Forms. A total of 25 questions were included and the survey was divided into two sections: (i) clinicians’ perceptions regarding the current use of scaffolds in the treatment of alveolar bony defects and (ii) clinical risk assessment of the newly developed biodegradable 3D-printed scaffold using the FMEA framework. The second section of the survey uses the FMEA framework where potential failure modes from the clinical use of the two biodegradable PCL scaffold (3D-printed meshes and porous blocks) were first identified based on literature review and the research team’s knowledge. The standards AS ISO 14971:2020, ISO/TR 24971:2020 and IEC 60812:2018 were used to guide this process. The clinicians were then required to assign each failure mode three separate ratings of 1–5 for severity (S), likelihood of occurrence (O), and likelihood of detection before adverse effects occur (D) according to a qualitative measurement scale provided (Table S1). The survey is attached in the appendix. The research was approved by The University of Queensland human ethics research committee (2021/HE000844).

### 2.2. Survey Validity and Reliability Assessments

Prior to using the questionnaire for information gathering from the target population, its validity and reliability was tested to evaluate its ability to measure what it is intended to measure and understand the reproducibility of its results.

The content validity of the designed questionnaire was assessed via the content validity index. A panel of five experienced clinicians consisting of periodontists and oral surgeons was asked to review the relevance of each question on a 4-point Likert scale as below:

1. Not relevant
2. Somewhat relevant
3. Relevant
4. Very relevant

If at least four out of five experts assigned the question a score of 3 or 4, this meant the content validity index was more than 80% and that question would remain in the questionnaire.

To evaluate the questionnaire’s reliability, its internal consistency was assessed by Cronbach’s alpha coefficient. The questionnaire was given to two independent periodontists at two different occasions with a week’s interval in-between. Alpha coefficient equal to or greater than 0.70 was considered satisfactory [19].

### 2.3. Participant Selection and Survey Distribution

The questionnaire was targeted towards dental specialists (predominantly periodontists) who practiced advanced surgical implant dentistry in Queensland, Australia. Surveys were sent to members of the Australian Society of Periodontology Queensland (ASPQ) branch, as well as prominent implant dentistry practitioners throughout the state. Using this targeted approach, a total of 46 survey invitations were sent to relevant clinicians that are most likely to be future users of this medical device.

### 2.4. Data Analysis

Once sufficient responses were gathered, all data were transferred to Microsoft Excel. Data were analyzed using statistical analysis features of Jamovi (Version 1.6.15). The responses to the first section of the survey were analyzed descriptively where frequencies and percentages were calculated for categorical variables. Chi-square test of independence was used for comparing some categorical variables to the outcome variables. Statistical significance was set at *p* < 0.05. The numerical responses to the second section of the survey were processed by multiplying the S, O, and D scores for each failure mode from each respondent to generate that particular failure mode’s risk priority number (RPN). The mean RPN and standard deviation of each failure mode were then calculated. High mean RPN would suggest that the failure mode has severe consequences, high probability of occurrence and/or low likelihood of detection [20]. Applying the Pareto Principle to risk analysis, 20% of potential failure modes contribute to 80% of the total RPN in a FMEA.18, 20. Thus, failure modes with RPNs in the top 20% will be considered high-priority for risk mitigation and will be highlighted to the product design and manufacturing teams so they can devise and implement appropriate safety measures.

## 3. Results

### 3.1. Survey Validity and Reliability

One out of 26 survey questions designed for this study was excluded as its content validity index was less than 80% based on the content validity assessment conducted. The questionnaire’s reliability in the form of its internal consistency was confirmed to be satisfactory with a Cronbach’s alpha coefficient of 0.84.

### 3.2. Demographics and Self-Rated Confidence in Using Scaffold Technologies

**Figure 1:**
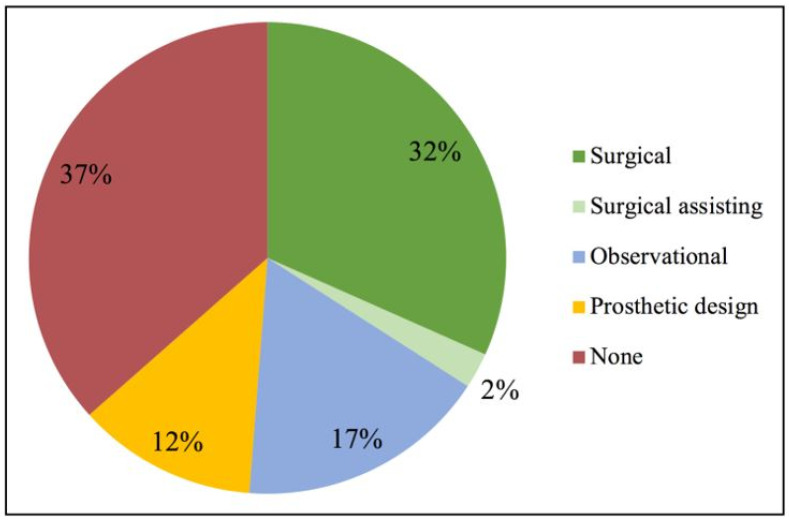
Participants’ level of experience with the use of personalized CAD/CAM scaffolds for horizontal and/or vertical bone augmentation.

There were 41 responses out of 46 distributed survey links, yielding an 89.1% response rate. Respondents reported varying degrees of experience with the use of personalized CAD/CAM scaffolds in the augmentation of horizontal and/or vertical bony defects (Figure 1). Thirty-four percent of the respondents were previously directly involved as a surgeon or surgical assistant in the placement of such scaffolds. The remaining 66% did not have prior experience of direct involvement in such procedures, but approximately half had indirectly participated as an observer or engaged in the prosthetic design of scaffolds.

A Chi-square test of independence was carried out to examine the relationship between clinicians’ experience with using personalized CAD/CAM scaffolds for treatment of horizontal and/or vertical bony defects, and their self-rated confidence in using biodegradable scaffolds for such procedures (Table S2). The relationship between these variables were found to not be significant; *χ*2 (df = 2, N = 41) = 0.308, *p* > 0.05. There was no significant disparity in self-rated confidence found between clinicians possessing surgical experience and clinicians who had none. Both experienced and non-experienced clinicians were mostly neutral or confident in the use of biodegradable scaffolds as a treatment modality.

### 3.3. Satisfaction with Current Titanium-Based Scaffolds

**Figure 2:**
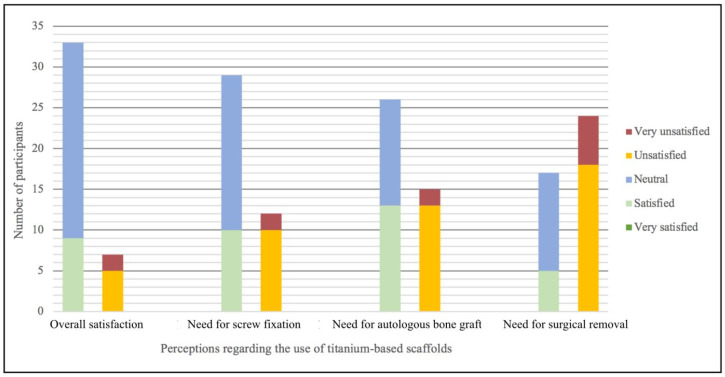
Participants’ satisfaction with various aspects of titanium-based scaffolds.

Majority of the clinicians surveyed were either satisfied or neutral about titanium-based scaffolds in general (Figure 2). With regards to the requirements of screw fixation and autologous bone grafts when using these scaffolds, most clinicians again expressed satisfaction or neutrality. Conversely, the need for post-treatment surgical removal of the nonresorbable titanium scaffold was a point of dissatisfaction for most of the clinicians surveyed. Moreover, none of the clinicians had indicated that they were ‘very satisfied’ with any of these requirements nor with the product overall. 

### 3.4. Perceived Performance and Advantages of 3D-Printed Biodegradable Scaffolds and Common Post-Operative Complications with the Use of Scaffolds

**Figure 3:**
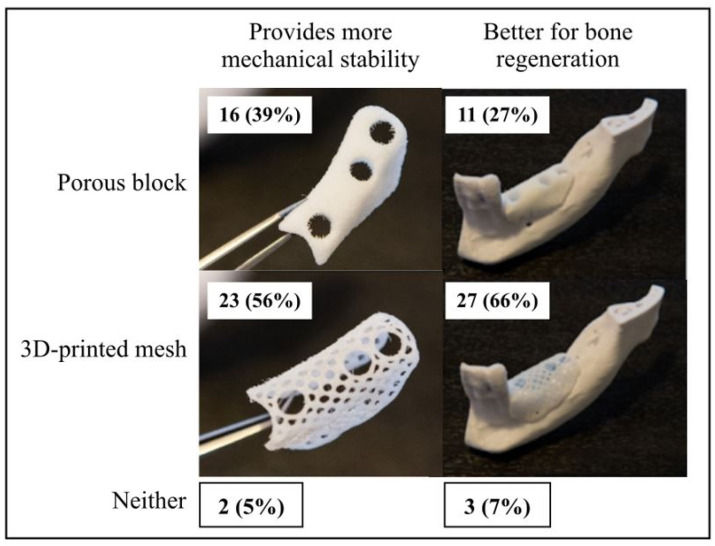
Comparison of clinical performance between the porous block and 3D-printed mesh.

Participants’ perceptions of the clinical performance of two scaffold types, porous block and 3D-printed mesh were compared. The qualities assessed were mechanical stability and facilitation of bone regeneration. More than half of the clinicians surveyed were of the opinion that 3D-printed meshes are superior to porous blocks in both aspects (Figure 3).

To further investigate which advantages of biodegradable 3D-printed scaffolds are most valued from a clinical perspective, participants were invited to choose as many options as they deemed significant from a selection of predetermined answers (Table 2). They were also given an option to contribute their own opinion, but no responses were gathered through this field. The most frequently chosen advantage was the absence of requirement for post-treatment surgical removal (24%), followed by the ability for prosthetic-guided planning (23%), high adaptability to a specific bony defect (22%), good soft tissue adaptation (17%), and finally acceleration of the surgical procedure (14%).

The participants were also asked to do the same with regard to the common post-operative complications that they encountered with the use of scaffolds (Table 2). Wound dehiscence (23%) was rated as the most common factor, followed by early graft exposure (22%), biomaterial resorption (18%), pain and discomfort (13%), poor stability of the newly formed bone once the scaffold is removed (13%), and site infection (10%).

### 3.5. FMEA Results

**Figure 4:**
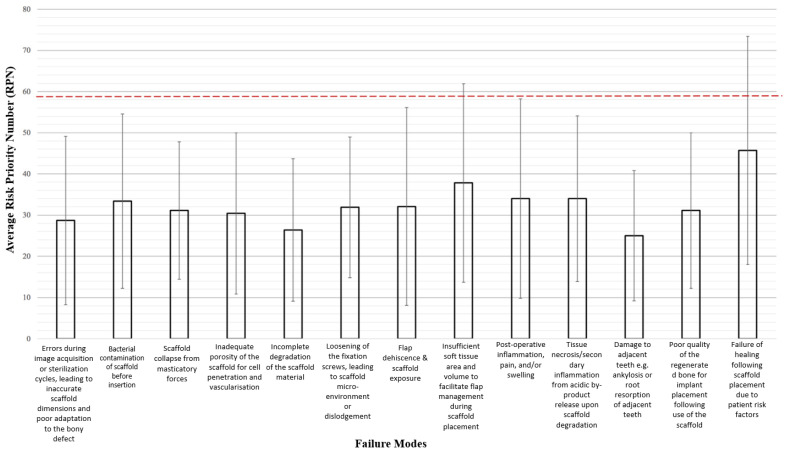
Mean RPNs of all 13 failure modes assess. Red dotted line indicates the threshold of risk priority number (RPN) at 59.

The average RPNs for the 13 failure modes are shown in Figure 4. There was a large variance within the RPN scores calculated for each failure mode, reflecting the differing opinions that clinicians had regarding the severity, occurrence, and detectability of the same failure mode. The highest average RPN is 45.7, and with a standard deviation of 27.7, the maximum RPN obtained was 73.4. Applying the Pareto Principle whereby 80% of the total RPN stems from the top 20% of failure modes, failure modes with RPN > 59 (top 20% of maximum RPN 73.4) are considered to be high-priority failure modes.

### 3.6. High-Priority Failure Modes

Two failure modes have mean RPNs that exceed the aforementioned threshold and are thus designated as high priority for the purposes of this risk analysis. Failure of healing following scaffold placement due to patient risk factors was assigned the highest mean RPN (45.7 ± 27.7), followed by insufficient soft tissue area and volume to facilitate flap management during scaffold placement (37.8 ± 24.1). Coincidentally, the highest average S (3.9 ± 1.0), O (3.4 ± 1.0), and D (3.3 ± 1.0) scores across all the failure modes came from the same failure mode, which also had the highest mean RPN that is healing failure due to patient risk factors.

## 4. Discussion

This study examined clinicians’ perceptions regarding the current use of scaffolds in the treatment of alveolar bony defects, in addition to performing clinical risk assessment of the newly developed biodegradable 3D-printed COR3 scaffold using the FMEA framework. Most clinicians surveyed were either satisfied or neutral about existing titanium-based scaffolds, as well as the need to use screw fixation and autologous bone grafts. However, higher dissatisfaction compared to satisfaction rates could be seen with the need to surgically remove titanium-based scaffolds. It thus appears that the need for surgical removal remains a major drawback of the current titanium-based scaffold technology. Xie et al. in 2020 reported such shortcomings of a second-stage surgery which causes additional trauma to the patient [21]. The need for postoperative systemic antibiotics after surgical removal of the scaffold also presents another disadvantage to the patient. These findings thus highlight the current dissatisfaction with titanium-based scaffolds and the need for future scaffold technologies to be biodegradable. In addition, maintaining the health of peri-implant tissues is more challenging than natural teeth which makes the augmentation of the bone defects and control of the bone loss in the surrounding tissues more difficult [22]. As such, it is unsurprising that the clinicians hold positive perceptions on the potential of the biodegradable COR3 scaffold. The most frequently chosen advantage of biodegradable 3D-printed scaffolds according to the clinicians surveyed was the absence of requirement for post-treatment surgical removal (24%). More than half of the clinicians surveyed were also of the opinion that 3D-printed meshes are superior to porous scaffolds in terms of mechanical stability and facilitation of bone regeneration. This is likely due to the current advances in 3D-printed scaffold fabrication techniques which can produce constructs with satisfying mechanical strength by precisely controlling the overall geometrical design and porosity to facilitate bone regeneration [23].

Nevertheless, despite presenting great potential in improving bone augmentation procedures, biodegradable 3D-printed scaffolds are also subject to possible drawbacks and limitations. Carrying out risk analysis for the newly developed COR3 scaffold is crucial in bridging the gap between product development and the successful safe clinical translation of this product. Furthermore, no risk analysis study has previously been conducted on biodegradable 3D-printed scaffolds for large-volume alveolar bone augmentation.

Failure of healing due to patient risk factors was identified as the most critical failure mode in our study. This is consistent with the findings by Tonelli in 2011 which suggested an assessment of the patient’s metabolic conditions prior to regenerative therapy [2]. In particular, patients with systemic osteoporosis or osteopenia which can be linked to regressive states (post-menopause, senility) or secondary to osteomalacia, hyper-parathyroidism, and other metabolic disorders (e.g., diabetes) would experience higher percentage of complications and failures. Although these diseases are not absolute contraindications, modifiable risk factors should ideally be removed, patient lifestyle should be modified, and secondary forms of this disease should be treated prior to such regenerative therapies. This study therefore has highlighted the importance of patient case selection on the part of the prescribing clinician.

Another high-priority failure mode identified in this study is insufficient soft tissue area and volume to facilitate flap management. As reported by Xie et al., the most important factors that may limit regeneration are soft tissues-especially in atrophic sites where the proximity to muscle insertions and the lack of keratinized mucosa influence the flap mobilization and therefore increase the risk of dehiscence [21]. Therefore, it might be important for CAD/CAM technology to take the soft tissue volume into account when designing the scaffold to ensure the perfect fit to the site. The thickness of the scaffold should also ideally allow some flexibility that reduces the risk of soft tissue dehiscence while maintaining sufficient rigidity and mechanical stability.

There are some limitations to this study. The dataset collected was relatively small with only 41 responses. This small sample size inherently leads to a higher variability in responses. Moreover, two-thirds of participants did not have surgical experience in scaffold placement due to limited availability of approved commercially produced 3D-printed scaffolds. These clinicians’ responses were likely based on theoretical knowledge or observational learning rather than actual past encounters of these complications. Therefore, the failure modes designated as top priority may not be fully reflective of clinical practice. Furthermore, RPN is calculated as S×O×D and thus different combinations of S, O and D scores can generate the same RPN number. This is problematic when a failure mode that is easily detected (low D) but has severe effects (high S) has the same RPN as a failure mode that is very difficult to detect (high D) and has less severe effects (low S). In this example, the former failure mode could be overshadowed by the latter when all the failure modes are being ranked solely in terms of RPN. Therefore, while it is important to prioritize the two failure modes that have yielded the highest average RPNs, the other failure modes should not be completely ignored. It might be useful to also prioritize the failure modes with high mean severity and/or occurrence ratings since those could represent a higher risk than failure modes with high detection ratings.

Considering the possible limitation discussed above about participants’ lack of surgical experience possibly influencing responses, future research in this domain may consider specifying surgical experience as one of the inclusion criteria for participating in the study. Moreover, the pool of surveyed clinicians mostly consisted of periodontists, and input may also be sought from oral-maxillofacial surgeons and general dentists who possess the relevant experience. In addition, although FMEA has been deemed to be the most suitable risk analysis method to implement at this stage of product development, complementing it with clinical trials would ensure that as many failure modes are identified and addressed as possible. Future studies should also look into risk management strategies in addition to risk analysis of this biodegradable patient-specific 3D-printed scaffold.

## 5. Conclusions

In conclusion, within the limitations of this study, there is evidence of positive perceptions of clinicians towards biodegradable 3D-printed scaffolds. Risk assessment conducted using the FMEA framework has revealed two high-priority failure modes that should be prioritized in risk mitigation for a safe clinical translation of the COR3 scaffold: (i) failure of healing due to patient risk factors and (ii) insufficient soft tissue area and volume to facilitate flap management.

## Figures and Tables

**Table 1:** Classification of medical devices based on their regulatory requirements based on The European Union Medical Device Regulation and Australia’s Therapeutic Goods Administration.

Medical Device Classification	Level of Potential Harm	Examples of Devices (Dental)
Class I	Lowest	Forceps, dental; Light, dental, polymerisation activator;
Class Is-sterile Class Im-measuring Class Ir-reusable	Low	Dental examination kit; Dental implant template set;
Class IIa	Low to Moderate	Artificial crown, custom-made, all ceramic; Surgical procedure kit, dental, non- medicated, reusable;
Class IIb	Moderate to High	Dental implant system; Dental implant, transgingival/intramucosal;
Class III	High	Dental bone matrix implant, synthetic -Q-Oss+

**Table 2:** Perceived advantages of biodegradable 3D-printed scaffolds and common post-operative complications with the use of scaffolds.

	Frequency
*Post-operative complications rated as most common in the treatment of horizontal and/or vertical bony defects* Pain and discomfort (N, %) Suture dehiscence (N, %) Early graft exposure (N, %) Sire infection (N, %) Biomaterial resorption (N, %) Poor stability of the newly formed bone once scaffold is removed (N, %)	15 (13%) 26 (23%) 25 (22%) 11 (10%) 20 (18%) 15 (13%)
*Advantages of 3D-preinted scaffolds rated as most significant in the treatment of horizontal and/or vertical bony defects* Prosthetic guided planning (N, %) High adaptability to a specific bony defect (N, %) Soft tissue adaptation (N, %) Acceleration of the surgical procedure (N, %) No need for surgical removal (N, %)	28 (23%) 27 (22%) 21 (17%) 17 (14%) 30 (24%)

## Data Availability

Not applicable.

## Supplementary Materials

The following supporting information can be downloaded at: https://www.mdpi.com/article/10.3390/ma15155442/s1, Table S1: Qualitative measurement scale used for the scoring of failure modes in the survey; Table S2: Frequency and percentages of self-rated confidence by level of experience.
