# Supplementary material for: A Clinical Risk Assessment of a 3D-Printed Patient-Specific Scaffold by Failure Modes and Effects Analysis
Source: Materials (Basel). 2022 Aug 8;15(15):5442. doi: 10.3390/ma15155442 (PMC9369557; doi:10.3390/ma15155442)
Supplement: Supplementary file 1 [file materials-15-05442-s001.zip › materials-1849407-supplementary.pdf]

materials MDPI

Table S1. Qualitative measurement scale used for the scoring of failure modes in the survey.

**Severity:** The magnitude of the consequences arising from the failure.				
Negligible (1)	Minor (2)	Moderate (3)	Severe (4)	Catastrophic (5)
The failure causes negligible effects.	The failure causes slight inconvenience and/or discomfort.	The failure causes temporary injury or impairment.	The failure causes permanent injury or impairment.	The failure causes life-threatening effects or patient death.
**Occurrence:** The likelihood of the failure occurring.				
Very low (1)	Low (2)	Moderate (3)	High (4)	Very high (5)
Failure is extremely unlikely.	Failure is unlikely.	Failure is probable.	Failure is likely.	Failure is nearly inevitable.
**Detectability:** The chances that the failure mode is detected before adverse consequences occur.				
Always detected (1)	Likely detectable (2)	Somewhat detectable (3)	Unlikely detection (4)	Undetectable (5)
Detection of the failure mode is almost certain.	Detection of the failure mode is likely.	Detection of the failure mode is somewhat likely.	Detection of the failure mode is unlikely.	Detection of the failure mode is nearly or completely impossible.

Table S2. Frequency and percentages of self-rated confidence by level of experience.

			**Level of confidence**	
	**Confident n (%)**	**Neutral n (%)**	**Unconfident n (%)**	**P-value**
Level of experience				
Surgical experience	4 (29)	8 (57)	2 (14)	0.857
No surgical experience	9 (33)	13 (48)	5 (19)	
